# Influence of the Big Five personality traits on intensive therapy adherence within people with aphasia

**DOI:** 10.3389/fpubh.2026.1793090

**Published:** 2026-03-30

**Authors:** Robert Darkow, Laura Plotho, Felix Muehlensiepen, Alexander E. Heidekum, Helmut Ritschl, Susann May

**Affiliations:** 1 Institute for Logopedics, JOANNEUM University of Applied Sciences, Graz, Austria; 2 AGEIS, Université Grenoble-Alpes, Grenoble, France; 3 Faculty of Health Sciences, Brandenburg Medical School Theodor Fontane, Center for Health Services Research, Rüdersdorf, Germany; 4 Department of Cardiology, Angiology and Intensive Care Medicine, German Heart Center of the Charité, Berlin, Germany; 5 Charité - University Medicine Berlin, Corporate Member of Freie Universität Berlin and Humboldt-Universität Berlin, Berlin, Germany

**Keywords:** aphasia, Big Five personality, communication, high-frequency speech therapy, therapy adherence

## Abstract

**Background:**

Guideline-based aphasia rehabilitation recommends intensive speech and language therapy, yet people with aphasia in German-speaking countries often receive substantially lower doses. Personality traits predict adherence in chronic disease, but their relevance for aphasia care is unclear. We examined associations between Big Five traits and therapy frequency and explored trait links to reported reasons for non-guideline-based care.

**Methods:**

From January to April 2024 we conducted a cross-sectional survey on patient-reported reasons for insufficient therapy. Overall, 260 individuals with aphasia participated; 243 completed the BFI-10. Median age was 62 years (range 20–88) and median aphasia duration 5.5 years. Therapy frequency was recorded categorically (none, monthly, weekly, twice weekly, 3 times weekly). Associations were tested using Spearman correlations and Mann–Whitney *U* tests.

**Results:**

Conscientiousness correlated positively with therapy frequency (*r* = 0.212, *p* < 0.001), whereas neuroticism (*r* = −0.115, *p* = 0.038) and openness (*r* = −0.138, *p* = 0.017) correlated negatively. Participants currently in therapy reported higher conscientiousness than those without therapy (*U* = 4472.5, *Z* = −2.961, *p* = 0.003). Agreeableness was higher among those endorsing “my therapist said I do not need therapy anymore” (*U* = 1839.5, *Z* = −2.175, *p* = 0.030). Neuroticism was higher among those endorsing “therapy was/is too exhausting” (*U* = 1327.5, *Z* = −2.238, *p* = 0.025). Conscientiousness correlated with perceived financial situation (*r* = 0.143, *p* = 0.027). No significant associations emerged for aphasia severity, age, or duration.

**Conclusion:**

Personality shows small but significant associations with therapy frequency and selected perceived barriers in aphasia rehabilitation. Given the cross-sectional, exploratory design, inferences are tentative. These effects were small and should be interpreted with caution. Results support incorporating personality into biopsychosocial models and motivate hypothesis-driven longitudinal studies.

## 1 Introduction

**Figure 1:**
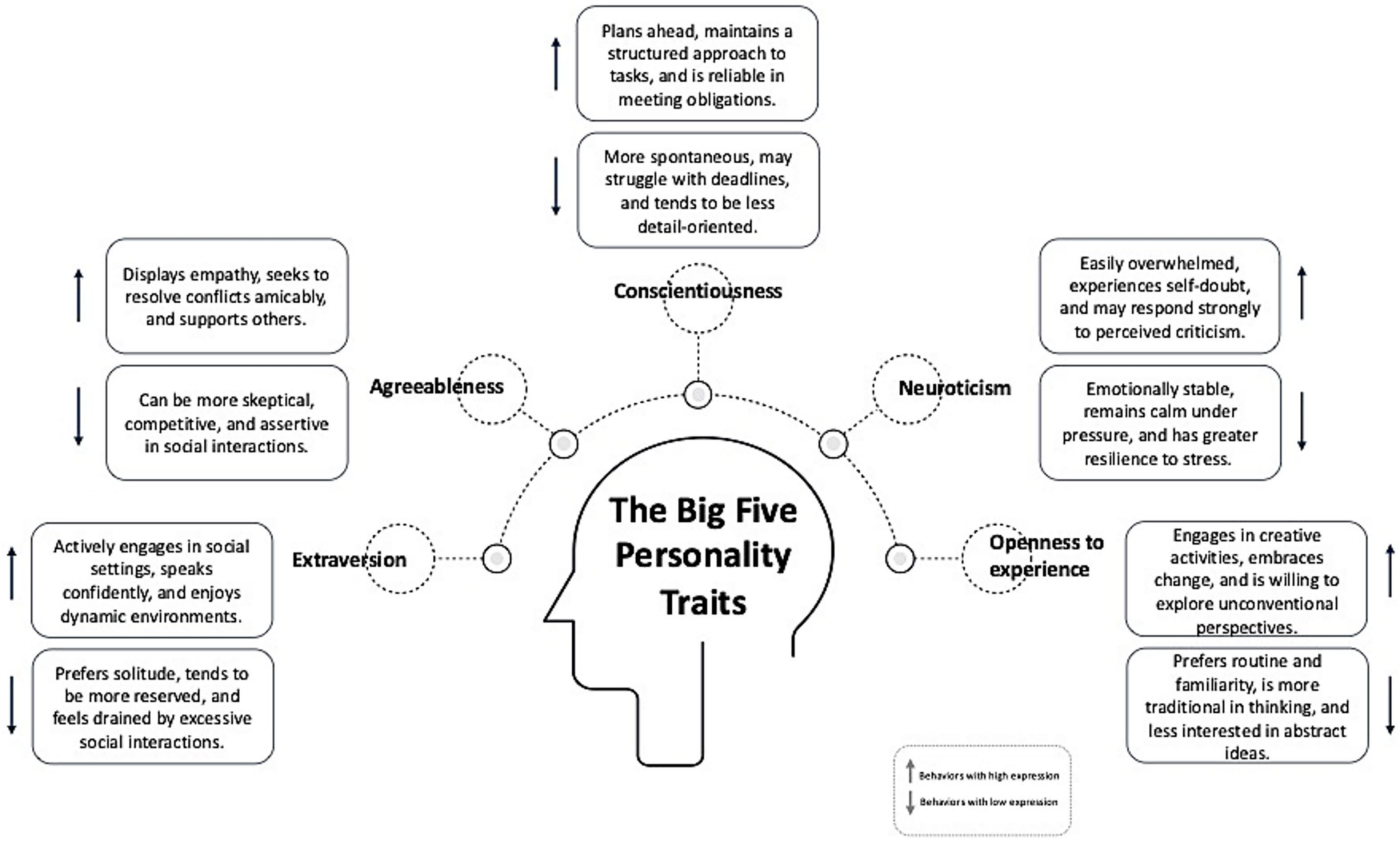
Visualization of the Big Five personality traits with examples of typical behaviors associated with high and low levels of each trait [based on John and Srivastava (2)]; own illustration.

Personality can be defined as relatively stable patterns of thoughts, feelings, and behaviors (1). A widely used framework is the Big Five model, which comprises the dimensions extraversion, agreeableness, conscientiousness, neuroticism, and openness to experience (2). These broad domains capture individual differences in social behavior, emotional regulation, and goal-directed behavior, and are commonly assessed using instruments such as the Big Five Inventory (3) (see Figure 1).

Personality, especially the Big Five, can predict a lot of aspects of people’s daily lives. For example, personality can influence job-related factors, election behaviors and health (4). Friedman et al. (5) showed that conscientiousness is associated with health and longevity, due to better health habits, like avoiding heavy drinking, smoking and less obesity. Conscientious people could be able to cope with stressful events better with proactive patterns of coping or pragmatically buying insurance for example. They also may maintain stable networks which predict health and longevity.

People with chronic illnesses must engage in treatment for the rest of their life, to be able to manage their disease. The World Health Organization defined adherence to long-term therapy as “the extent to which a person’s behavior – taking medication, following a diet, and/or executing lifestyle changes, corresponds with agreed recommendations from a health care provider.” (6). Some studies have examined the relationship between adherence to treatment and personality traits. A Swedish epidemiological study found that the traits neuroticism, agreeableness and conscientiousness influence adherence behavior, showing that neuroticism was negatively associated with adherence, while agreeableness and conscientiousness were positively associated with adherence (7). Another study found that medication adherence in the older adults was associated with higher mean scores in agreeableness and conscientiousness and non-adherence was associated with lower mean scores in neuroticism (8). Conscientiousness was associated with medication adherence in patients with Rheumatoid Arthritis (9), while high agreeableness was associated with better adherence to an exercise treatment in major depressive disorder (10). Also, mortality can be associated with personality traits. Christensen et al. (11) found that patients of chronic renal insufficiency and high scores in neuroticism and low scores in conscientiousness had a higher estimated mortality rate.

But not only the adherence of long-term treatment seems to be influenced by personality. Personality traits seem to be associated with adherence to short-term treatment, like antibiotics. Non-adherent patients score lower on agreeableness and conscientiousness and neuroticism seems to be a negative predictor of adherence behavior (7). Despite many studies associating Big Five personality traits with adherence in chronic diseases, to our knowledge, there has not been a study about people with aphasia and adherence to (long-term) treatment.

Aphasia is one of the most common disabilities following brain injury and represents a centrally caused language disorder that may occur after stroke, traumatic brain injury, or brain tumor. It affects several linguistic levels, leading to impairments in language production, comprehension, and written communication, with severity varying between individuals (12). Although the cognitive basis of an intended utterance is usually intact, verbalization is disturbed by missing or incorrect words or grammatical structures. On a broader level, aphasia restricts communication and social participation (13). These limitations affect professional and private life, reduce independence, and are often accompanied by decreased self-efficacy, psychosocial stress, and reduced well-being (14).

In addition to linguistic deficits, aphasia is associated with various behavioral and emotional changes. Consequently, individuals with aphasia experience an increased psychological burden, including significantly elevated levels of depression and anxiety. They tend to score lower on extraversion (i.e., they are more withdrawn) and higher on psychoticism (e.g., they are more irritable and hostile) on personality assessments (e.g., the Eysenck Personality Questionnaire, EPQ) compared to non-aphasic individuals (15). However, little is known about how broader personality and emotional factors influence recovery and rehabilitation outcomes. Gaining insight into these factors could lead to more personalized and effective therapeutic approaches for individuals with aphasia. Thus, the aim of our report is to examine personality traits of people with aphasia in relation to therapy frequency and therapy frequency satisfaction, as well as reasons for non-guideline-based speech therapy.

The present study is part of a broader investigation into barriers to guideline-based speech and language therapy (16) from the perspective of people with aphasia. It is well established that individuals with aphasia in Germany do not receive therapy in accordance with clinical guidelines (17), with therapy provision amounting to only one-twentieth of the recommended 10 h per week. To date, the reasons for this deficit have not yet been systematically investigated. A survey by Schönle and Lorek (18) among speech and language therapists indicated that limited therapy engagement was often attributed to the patients themselves. Recruitment challenges for innovative therapeutic approaches have also been noted (19). Initial studies suggest that personality traits may influence therapy outcomes. For instance, higher levels of negative affectivity as measured by instruments such as the PANAS were predictive of poorer progress in speech-language therapy. Overall, psychological and neurocognitive factors accounted for approximately 15% of the variance in therapy progress, beyond the effects of aphasia severity (20). These findings lead to the research questions of the current study: (1) Is there a relationship between specific Big Five personality traits and therapy frequency in individuals with aphasia? (2) Is there a relationship between specific Big Five personality traits and reasons for non-guideline-based speech therapy in individuals with aphasia?

## 2 Methods

### 2.1 Sample

This study reports on findings from the analysis of data collected as part of a cross-sectional, self-completed, online survey of German and Austrian people with aphasia from January 2024 to April 2024. The study was approved by the data protection officer and the ethics committee of the Medical University of Graz, Reference ID (EK-Number): 35-389 ex 22/23. The inclusion criteria for participants were: (1) aphasia, (2) age ≥18, (3) native German speaker, (4) participation in speech therapy in the past, and (5) willingness to participate in the study. The survey was developed based on the results of a previous qualitative study (16) with 21 people with aphasia, which explored the reasons for non-utilization of speech therapy.

### 2.2 Questionnaire development

The questionnaire was designed in an aphasia-friendly format. This included the use of simple and clear wording, short sentences, and a clear and consistent layout. In addition, supportive pictograms were incorporated. Complex or abstract content was minimized where possible, and response formats were kept simple and visually well structured. The questionnaire was pretested with 11 healthy individuals and 5 people suffering from aphasia to ensure survey comprehensibility and clarity. The questionnaire was revised in various feedback rounds. In total, the questionnaire was revised 6 times until no more uncertainties appeared among the test persons. The final questionnaire was administered using SoSci Survey, a web-based survey tool (21). To ensure data quality and avoid duplicate participation, the survey system checked whether participants accessed the survey multiple times. Technical measures such as cookies or temporary IP checks may have been used for this purpose. No personal data was stored. A paper pencil version was also created. The final questionnaire was divided into the following sections: (1) Using of speech therapy. (2) Information on the frequency of speech therapy. (3) Satisfaction with the frequency of speech therapy (another publication on these results is planned and is not part of this paper). (4) Information on reasons for not using speech therapy or using it too little speech therapy. (5) Wishes for speech therapy and the care system (another publication on these results is planned and is not part of this paper). (6) Information on personality characteristics. (7) Sociodemographic information.

The final questionnaire is presented in Supplementary material S1. The survey was conducted over a 4-month period between January 2024 to April 2024 in accordance with the Checklist for Reporting of Survey Studies (CROSS) [(22); Supplementary material S2]. The participants received no incentives for their participation.

### 2.3 Recruitment

**Figure 2:**
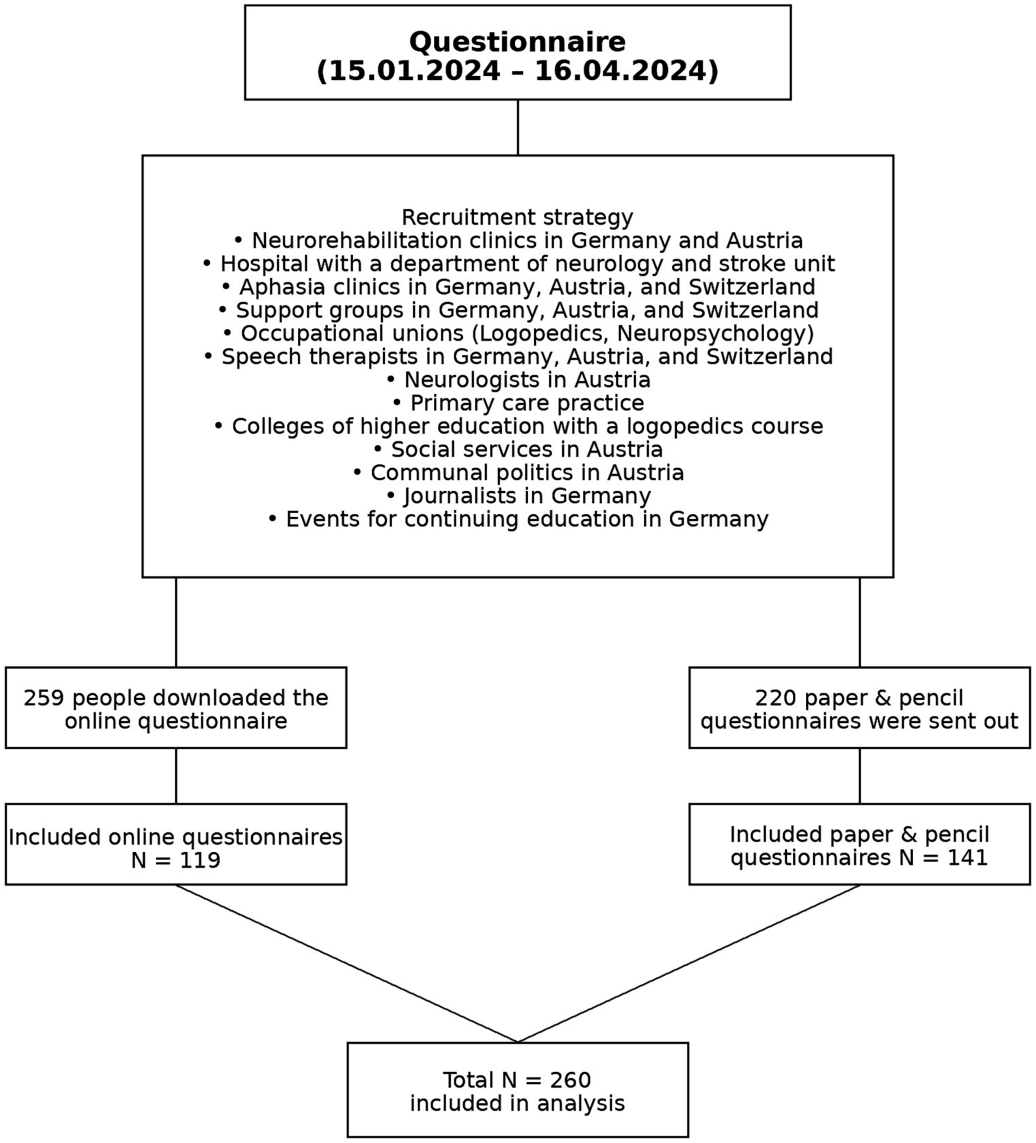
Flowchart of the recruitment and inclusion process for the questionnaire study.

Clinics with a neurology department, neurorehabilitation clinics, neurologists, speech therapist, primary care practices, colleges of higher education with a logopedics course, communal politics, support groups, social services, journals, occupational unions and events for continuing education were all part of recruitment (see Figure 2). The return rate of the paper-pencil questionnaire was about 64%. The online questionnaire was opened 946 times and downloaded 259 times. We included questionnaires where the sections “information on reasons for not using speech therapy or using too little therapy” and “wishes for speech therapy and the care system” were filled out. We were able to include 119 online questionnaires. Overall, 260 questionnaires were included in the data analysis. Of the 260 participants included in the study, 243 completed the personality questionnaire (BFI-10). Analyses involving personality traits were therefore based on this subsample. Both the paper–pencil and online versions of the questionnaire were distributed exclusively via clinical and care institutions to ensure that participants with aphasia had access to communicative support. Institutions were instructed to provide assistance with comprehension and response formulation when necessary. Individuals with aphasia were not recruited directly. In addition, information was also collected on who completed the questionnaire. 56.8% (*n* = 137) of the questionnaires were completed by the aphasic individuals themselves, 19.1% (*n* = 46) were completed by family members as proxies, and 24.1% (*n* = 58) were completed with support from proxies (with the aphasic individual present). Proxy participation increased significantly with the severity of aphasia [11.8% mild, 38.9% moderate, 63.0% severe; χ^2^(2) = 27.55, *p* < 0.001]. This procedure was intended to support adequate understanding of the items, particularly for participants with more severe language impairments.

### 2.4 Measures

To measure personality, the BFI-10 (23) German version was used. The BFI-10 is a short version of the BFI (3). The BFI-10 is suited for assessments of personality for surveys with multiple matters, because it only contains 10 items for assessing the Big Five personality domains. Each Big Five personality domain is measured with two items, with one item being negatively coded. The BFI-10 uses a 5-point response scale, ranging from 1 (strongly disagree) to 5 (fully agree). Current therapy frequency was assessed with the question: “How often do you currently attend speech and language therapy?” Response options were: Not currently in therapy, once per month, once per week, twice per week or 3 times per week. Current therapy frequency was assessed as the number of sessions actually attended rather than prescribed. In Germany and Austria, therapy frequency is partly determined by medical prescriptions and health insurance regulations and may therefore not solely reflect patient adherence. Therapy frequency satisfaction was operationalized using the item: “I am satisfied with the frequency of therapy.” Responses were recorded on a five-point Likert scale: Fully agree, agree, partially agree, rather disagree, and do not agree at all. The reasons for not participating in speech and language therapy were based on the results of the qualitative previous study (16).

### 2.5 Statistical analysis

The negative coded items were recoded and the means of the dimensions were calculated. The statistics software SPSS, version 29, was then used for further statistical analysis.

Spearman’s correlation was used to analyse the associations between the Big Five personality traits and therapy frequency, as well as therapy frequency satisfaction.

Furthermore, as the dependent variables were not normally distributed according to the Kolmogorov–Smirnov and Shapiro–Wilk tests (all *p* < 0.001), and group sizes were unequal, non-parametric Mann–Whitney *U* tests were used to analyse the mean differences in personality traits and reasons for receiving no therapy or non-guideline-based therapy.

Additionally, ANOVA, Spearman and Pearson correlations, as well as Mann–Whitney-*U* tests, were conducted to identify differences in personality traits and sociodemographic characteristics.

The significance level was set at *p* < 0.05. Due to the exploratory nature of this study, no correction was made for multiple comparisons; additionally, trend-level findings (0.05 ≤ *p* < 0.10) are reported separately from the main results.

## 3 Results

The study included 260 participants with aphasia, 243 participants filled out the personality questionnaire. The median age was 62 years (min: 20 years, max: 88 years) and the median duration of aphasia 5.5 years (detailed sociodemographic information please see Table 1).

**Table 1:** Sociodemographic data.

Characteristics	Participants (*n* = 243)	
	*n*	%
1. Gender		
Female	106	43.6
Male	125	51.4
Missing	12	4.9
2. Self-perceived severity of aphasia		
Mild	33	13.6
Moderate	117	48.1
Severe	77	31.7
Missing	16	6.2
3. Place of residence		
Village	60	24.7
Small town	70	28.8
Large city	99	40.7
Missing	14	5.8
4. Education level		
No vocational training	16	6.6
Completed vocational training	141	58
Academic degree	71	29.2
Missing	15	6.2

### 3.1 Main findings (*p* < 0.05)

Correlations between personality traits and therapy frequency/therapy frequency satisfaction. The results showed a significant correlation between therapy frequency and the personality trait consciousness (*r* = 0.212, *p* < 0.001), neuroticism (*r* = −0.115, *p* = 0.038) and openness (*r* = −0.138, *p* = 0.017). People who have higher scores on consciousness are more likely to receive more therapy. People who have higher scores on neuroticism are more likely to receive less therapy, as well as higher scores on openness. No other significant correlations between personality traits and therapy frequency as well as therapy frequency satisfaction were found (results can be found in Supplementary material S3).

To analyse the robustness of the found bivariate associations, an ordinal logistic regression model was performed, including all five personality traits and key covariates (age, aphasia duration/severity, professional training and financial situation; *N* = 228). The model showed excellent fit (−2LL reduction χ^2^(10) = 72.5, *p* < 0.001; Nagelkerke *R*^2^ = 0.292). Higher conscientiousness (B = 0.388, *p* = 0.016, OR = 1.47) and lower openness (B = −0.276, *p* = 0.028, OR = 0.76) independently predicted greater therapy frequency. Neuroticism attenuated (B = −0.165, *p* = 0.215), while severity (OR = 2.56), duration (OR = 0.94), and age (OR = 0.97; all *p* < 0.01) emerged as further predictors.

Group differences in personality traits and reasons for receiving no therapy or non-guideline-based therapy. A Mann–Whitney-*U* test showed that people with aphasia who currently have speech therapy have higher scores on conscientiousness than those who have no therapy (*U* = 4472.500, *Z* = −2.961, *p* = 0.003).

Mann–Whitney-*U* tests showed differences between reasons for not receiving guideline-based therapy and personality traits. There was a significant difference in the scores of agreeableness and the reason “my therapist said I do not need therapy anymore” (*U* = 1839.500, Z = −2.175, *p* = 0.030). In addition, there was a significant difference in the scores of neuroticism and the reason “therapy was/is too exhausting” (*U* = 1327.500, Z = −2.238, *p* = 0.025).

Associations between personality traits and sociodemographic characteristics. Spearman correlation found a significant association between the financial situation and the personality trait conscientiousness (*r* = 0.143, *p* = 0.027). People with higher scores in conscientiousness are more likely to feel that they are doing well financially. Furthermore, Mann–Whitney-*U* test showed that people with aphasia in the chronic stage have significantly higher scores in openness than people with aphasia in the post-acute phase (*U* = 2,206, *Z* = −2.694, *p* = 0.007). There have been no significant results regarding personality traits and sociodemographic variables severity, age and duration of aphasia.

### 3.2 Trend-level findings

In addition to the above-described results, the following trend-level findings (0.05 ≤ *p* < 0.10) were found:

Mann–Whitney *U* tests revealed several associations at trend level between personality traits and reasons for insufficient therapy frequency. Extraversion tended to differ between individuals citing “I have no time for speech therapy” (*U* = 1501.000, *Z* = −1.936, *p* = 0.053) and others, with the “no time” group showing lower mean ranks. Additionally, extraversion also tended to differ between individuals citing “my aphasia is not severe enough” and others (*U* = 2044.500, *Z* = −1.795, *p* = 0.073), with the “my aphasia is not severe enough” group showing higher mean ranks.

The trends for the personality trait of agreeableness were found to be almost identical. People with aphasia who cited “I have no time for speech therapy” (*U* = 1578.500, *Z* = −1.669, *p* = 0.095) or those who cited “my aphasia is not severe enough” (*U* = 2028.500, *Z* = −1.857, *p* = 0.063) as the reason for insufficient therapy frequency showed higher mean ranks than others.

Individuals citing “I have had aphasia for a long time” as a reason for insufficient therapy showed lower mean ranks in conscientiousness than others (*U* = 3550.000, *Z* = −1.958, *p* = 0.050).

For neuroticism, individuals citing “my therapist said I do not need therapy anymore” (*U* = 1925.000, *Z* = −1.886, *p* = 0.059) showed lower mean ranks than others and individuals citing “I have no time for speech therapy” (*U* = 1578.00, *Z* = −1.661, *p* = 0.097) showed higher mean ranks than others.

Results from the ANOVA indicate significant differences in conscientiousness across levels of professional training [*F*(2, 240) = 3.06, *p* = 0.049]. Although the Bonferroni *post hoc* test did not reveal statistically significant pairwise differences, there was a trend toward higher conscientiousness among individuals with a university degree compared to those without professional training (*p* = 0.062).

## 4 Discussion

This report examined associations between personality traits, therapy frequency, satisfaction, and reasons for non-guideline-based therapy in people with aphasia. A positive correlation was found between therapy frequency and conscientiousness, and negative associations with neuroticism and openness. An additional sensitivity analysis (e.g., logistic ordinal regression) revealed the robustness of the associations between therapy frequency and conscientiousness, as well as openness. However, the effect of neuroticism was fully attenuated. Overall, these findings suggest that individuals high in conscientiousness may be more inclined to adhere to therapy guidelines due to a preference for structure, while lower engagement in therapy among individuals who score highly on the openness dimension may be related to preferences for novelty, autonomy, and flexible contexts rather than routine and structure. Standardized, guideline-based speech and language therapy, which is typically characterized by frequent and repetitive sessions, may be less aligned with these tendencies.

However, this interpretation should be considered with caution, as the present data do not allow conclusions about the underlying mechanism, therefore this interpretation represents only one possible explanation of the observed association. From a broader perspective, individuals high in openness may also differ in their patterns of healthcare utilization. For example, they may be more inclined to seek alternative or complementary treatment approaches, including interventions that are not part of standard guideline-based care or not captured in the present survey. In addition, highly structured and repetitive therapy protocols may be perceived as less engaging or meaningful, which could influence sustained participation. It is therefore likely that the observed negative association between openness and therapy frequency reflects a combination of individual preferences, perceptions of therapy, and structural aspects of the healthcare system, rather than a single underlying mechanism. Therefore, interventions that promote self-efficacy and autonomy could enhance engagement among highly open individuals.

People with higher agreeableness more often cited “My therapist said I don’t need therapy anymore,” suggesting a tendency to accept external authority. Higher neuroticism was linked to citing therapy as “too exhausting,” reflecting increased emotional vulnerability. Furthermore, at a trend level, individuals high in extraversion or agreeableness frequently stated lack of time or perceived mild symptoms as reasons for insufficient therapy, while high conscientiousness was linked to the belief that therapy was unnecessary due to the long duration of living with aphasia. These preliminary patterns imply that personality traits shape attitudes and perceived barriers to therapy adherence.

Sociodemographic findings aligned with previous research: people with aphasia and a university degree scored higher in conscientiousness, which also correlated with better financial status—consistent with studies showing links between conscientiousness, career success, and income (24).

Research on the Big Five in aphasia therapy adherence is scarce. Votruba et al. (20) investigated affective symptoms and their predictive value for speech therapy outcomes and found that higher levels of negative affectivity were associated with poorer therapy progress. In the present study, higher neuroticism was associated with perceiving therapy as exhausting, suggesting that emotional burden may be associated with reduced engagement in therapy. Neuroticism is commonly understood as reflecting a tendency toward negative emotional states such as anxiety, tension, and distress. In this context, affective processes may represent a relevant explanatory framework. Affectivity, often conceptualized as positive and negative affect, describes relatively stable emotional tendencies that have been linked to health-related behaviors and treatment adherence in other populations. However, affectivity was not directly assessed in the present study, and therefore its role cannot be determined. These interpretations should be considered exploratory. Future research should include direct measures of affective states to clarify their contribution to therapy engagement and to better understand the relationship between personality traits such as neuroticism and rehabilitation outcomes.

Watson and Clark describe NA as a dispositional mood dimension marked by chronic distress and negative self-concept (25). Such emotional states regardless of external stressors may interfere with the effectiveness of therapy. Given that stress hinders learning (26–28), therapists should recognize the emotional profiles of patients with aphasia and aim to reduce stressors in therapy environments to improve outcomes. Although affectivity was not directly measured in this study, it is conceptually linked to neuroticism, which was found to have a significant association with therapy adherence. Future research should explicitly assess affective states and their impact on therapy adherence to enable more tailored interventions for individuals with aphasia.

The present findings should be interpreted in light of a well-documented discrepancy between guideline-recommended and actually delivered aphasia therapy. Studies indicate that people with aphasia often do not receive therapy at the recommended intensity, with actual provision falling substantially below guideline recommendations. This gap in care represents not only a clinical issue but also a relevant public health concern. The present results further suggest that this underutilization cannot be explained solely by structural factors, but may also be related to individual differences in health behavior. In particular, the observed associations between personality traits and therapy frequency indicate that individuals with different personality profiles may engage differently with available healthcare services. From a public health perspective, this implies that the utilization of rehabilitation services is determined not only by access to care, but also by individual dispositions that influence motivation, emotional regulation, and preferences. Focusing exclusively on structural barriers may therefore be insufficient to address the underutilization of aphasia therapy.

### 4.1 Practical implications

Within a multifactorial model of therapy uptake, the present Big Five findings in aphasia have clinically relevant—yet clearly bounded—implications: personality may add a further layer shaping engagement and adherence-related behavior, but with small effect sizes and limited clinical variance explained, so it should be interpreted as a contributing factor rather than a primary determinant; this is consistent with reports that global personality measures are generally weak adherence predictors (29). Therapy uptake—and especially guideline-concordant intensity—of speech-language therapy for aphasia is best conceptualized as a multifactorial outcome of clinical “need,” reflected in impairment severity, overall functional burden, and comorbidity; contextual access and system constraints shaped by capacity, transport, costs or reimbursement, and time or administrative demands; and psychosocial determinants, most notably social support alongside treatment beliefs and expectancies, consistent with established adherence and healthcare utilization frameworks (30–32). In chronic conditions, depressive symptoms robustly increase the odds of non-adherence (33). Across rehabilitation settings, practical “enabling” barriers such as travel, scheduling and financial burden recur and often dominate day-to-day feasibility, and organisational factors may outweigh sociodemographic predictors (34, 35). In aphasia, qualitative evidence similarly attributes non-participation in high-frequency, guideline-based therapy primarily to health-system and organisational barriers, notably workforce shortages, bureaucratic or coordination burden, and limited availability, alongside practical access problems such as costs and distance, whereas condition-related factors are typically contributory rather than sufficient explanations (16). Psychosocially, treatment beliefs, with perceived necessity versus concerns, and expectations are well-established correlates of adherence-relevant behavior, and social support is a recognized antecedent of adherence processes (36–38). Clinically, the implication is not a new primary determinant, but an additional tailoring lever within person-centered care: personality-informed adaptations such as more structured schedules, frequent feedback, and supportive coaching, together with flexible formats aligned with stress coping, time management and perceived self-efficacy, may help anticipate barriers early without downplaying structural constraints (37, 39–41).

### 4.2 Limitations

Despite the interesting findings, this study has several limitations. Firstly, the assessment of personality traits relied on self-reports or proxy ratings, which may have affected the validity of the results due to the communication difficulties that are often present in individuals with aphasia. Another limitation concerns the use of the ultra-short BFI-10 to assess personality traits in people with aphasia. While this shortened version of the BFI-44 has been shown to have robust psychometric properties (e.g., mean part-whole correlation r = 0.83 with the full BFI-44 version, mean retest stability coefficients r = 0.75, clear five-factor structure, etc.; (23, 24)), it was designed primarily for large-scale surveys under severe time constraints, providing only an approximate indication of the Big Five traits. Furthermore, and most importantly, the BFI-10 has not yet been formally validated in populations with aphasia, and its psychometric properties have mainly been established in cognitively and linguistically unimpaired adults. Consequently, the personality scores reported here should be interpreted as approximate rather than precise estimates of traits, and future studies should use more comprehensive, aphasia-adapted personality measures. Finally, the cross-sectional nature of the study also prevents any conclusions about causality. It remains unclear whether personality traits influence therapy adherence or whether prolonged therapy experiences shape certain personality characteristics. Potential confounding factors such as social support, cognitive impairments, or depressive symptoms were not controlled for, even though these are known to influence adherence behavior.

## 5 Conclusion

Personality traits appear to influence how frequently individuals with aphasia participate in speech therapy, with neuroticism showing a particularly strong association. To our knowledge, this is the first study to examine the relationship between personality characteristics and adherence to therapy in this population. Future research should build on these findings by using longitudinal designs to clarify causal mechanisms, as well as conducting qualitative investigations to explore the subjective experiences that underlie therapy engagement. Further studies should incorporate more comprehensive personality assessments, consider relevant psychosocial factors such as depression and social support, and evaluate the effectiveness of personalized therapeutic approaches. As these relationships are unlikely to be driven by personality alone, subsequent work should examine the contribution of affectivity, particularly negative affect, and investigate whether reducing negative affectivity enhances adherence and improves treatment outcomes.

## Data Availability

The raw data supporting the conclusions of this article will be made available by the authors, without undue reservation.
