# Supplementary material for: Influence of the Big Five personality traits on intensive therapy adherence within people with aphasia
Source: Front Public Health. 2026 Mar 30;14:1793090. doi: 10.3389/fpubh.2026.1793090 (PMC13071038; doi:10.3389/fpubh.2026.1793090)
Supplement: Supplementary file 1 [file Supplementary_file_1.docx]

Dear participants,

welcome to our study **„AWARE-What do people with aphasia want?“**. Guidelines for aphasia therapy suggest that 6-10 hours of speech therapy per week are necessary for significantly improving aphasia. However, this is not implemented in reality. We would like to understand why people with aphasia receive insufficient or no speech therapy at all. What do individuals with aphasia wish for regarding speech therapy and politics?

This study is conducted by the Center for Health Services Research at the Brandenburg Medical School and FH JOANNEUM Graz. The study is funded by the Hannelore Kohl Foundation and has been approved by the Ethics Committee of the Medical University of Graz.

Your responses will be treated confidentially. Personal information will remain anonymous, no conclusions can be drawn about your identity.

The survey will take approximately 30 minutes.

The questionnaire can also be filled out by relatives of individuals with aphasia.

The questionnaire begins on the next page.

Icons by Icons8 [www.icons8.com](http://www.icons8.com)

| 🞏 I have aphasia.  🞏 I am a family member or a caregiver of someone with aphasia.  🞏 I am a family member or a caregiver of someone with aphasia and I will complete this questionnaire together with the person affected by aphasia. |
| --- |

| Do you have speech therapy **at the moment**? | | | | | | |  | | | | | |
| --- | --- | --- | --- | --- | --- | --- | --- | --- | --- | --- | --- | --- |
| 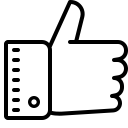Yes  🞏 | | | | | | 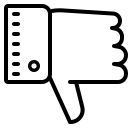No  🞏 | | | | | | |
|  | | | | | |  | | | | | | |
| How often do you go to speech therapy? | | | | | | | | | | | | |
| **No** therapy  🞏 | **Once** a  **month**  🞏 | | **Once** a  **week**  🞏 | | | | | **Twice** a  **week**  🞏 | | | **Three times** a **week**  🞏 | |
|  | How **long** does speech therapy last? | | | | | | | | | | | |
|  | **30** minutes  🞏 | | | | **45** minutes  🞏 | | | | | **60** minutes  🞏 | | |
|  | | | | | | | | | | | | |
| I am **satisfied** with the **amount** of speech therapy I am **receiving**. | | | | | | | | | | | | |
| **Fully** agree | | **Agree** | | **Partially**  agree | | | | | **Disagree** | | | **Fully**  disagree |
| 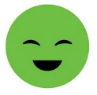🞏 | | 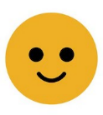🞏 | | 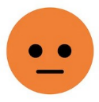🞏 | | | | | 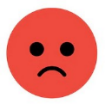 🞏 | | | 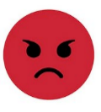 🞏 |
|  | |  | |  | | | | |  | | |  |
| I know where I can get information about aphasia.  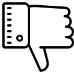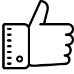  🞏 No 🞏 Yes | | | | | | | | | | | | |
|  | | | | | | | | | | | | |
| Do you want to say something regarding this topic?   \|  \| \| --- \| \|  \| \|  \| \|  \| | | | | | | | | | | | | |

Do the following reasons why you do not receive or receive insufficient speech therapy apply to you?

Please choose between yes⌧ or no ⌧.

| I do not receive any or sufficient speech therapy, because **speech** **therapists** nearby **only treat children**. | 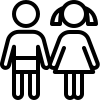 | Yes  🞏 | No  🞏 |
| --- | --- | --- | --- |
| I do not receive any or sufficient speech therapy, because my **speech therapist said**, that I do **not longer need** therapy. | 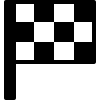 | Yes  🞏 | No  🞏 |
| I do not receive any or sufficient speech therapy, because the speech therapist **does not suit me.** | 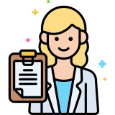 | Yes  🞏 | No  🞏 |
| I do not receive any or sufficient speech therapy, because I **did not like** the **goals** of speech therapy. | 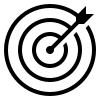 | Yes  🞏 | No  🞏 |
| I do not receive any or sufficient speech therapy, because my speech therapist has gone on **maternity leave**, and I **do not have** a **replacement**. | 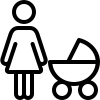 | Yes  🞏 | No  🞏 |
| I do not receive any or sufficient speech therapy, because my speech therapist **retired**, and I **do not have** a replacement. | 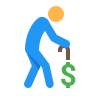 | Yes  🞏 | No  🞏 |
| I do not receive any or sufficient speech therapy, because speech therapy is **too exhausting**. | 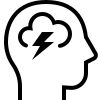 | Yes  🞏 | No  🞏 |
| I do not receive any or sufficient speech therapy, because speech therapy is **too expensive**. | 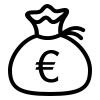 | Yes  🞏 | No  🞏 |
| I do not receive any or sufficient speech therapy, because I **do not have** the **time** for speech therapy. | 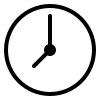 | Yes  🞏 | No  🞏 |
| I do not receive any or sufficient speech therapy, because there is **no speech therapist nearby.** | 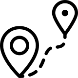 | Yes  🞏 | No  🞏 |
| I do not receive any or sufficient speech therapy, because the **practice is not accessible for people with disabilities.** | 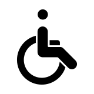 | Yes  🞏 | No  🞏 |
| I do not receive any or sufficient speech therapy, because my aphasia is not severe enough. | 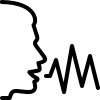 | Yes  🞏 | No  🞏 |
| I don't receive any or sufficient therapy because I've had aphasia for a long time. | 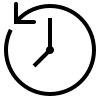 | Yes  🞏 | No  🞏 |
| I do not receive any or sufficient speech therapy, because I **do not have anyone to support me**. | 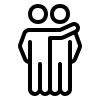 | Yes  🞏 | No  🞏 |
| I do not receive any or sufficient speech therapy, because it is **stressful** to **constantly get prescriptions** from the doctor. | 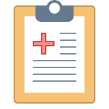 | Yes  🞏 | No  🞏 |
| I do not receive any or sufficient speech therapy, because the doctor **no longer prescribes speech therapy**. | 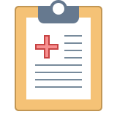 | Yes  🞏 | No  🞏 |
| I do not receive any or sufficient speech therapy, because I **do not make any progress** anymore. | 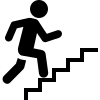 | Yes  🞏 | No  🞏 |

The following questions concern your wishes regarding the care of individuals with aphasia.

Please answer **Yes ⌧** or **No ⌧**.

| 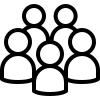  I wish for **more group therapy**. | | | |
| --- | --- | --- | --- |
| Yes 🞏 | How **important** is it to you? | | |
|  | **Very** important  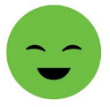🞏 | **important**  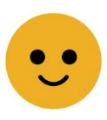🞏 | **Less** important  🞏  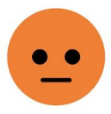 |
| No 🞏 | | | |

| 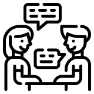  I wish for **more** speech therapy **sessions** during a **week**. | | | |
| --- | --- | --- | --- |
| Yes 🞏 | How **important** is it to you? | | |
|  | **Very** important  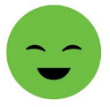🞏 | **Important**  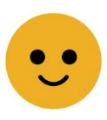🞏 | **Less** important  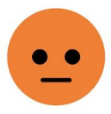🞏 |
| No 🞏 | | | |

| 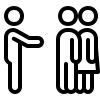  I wish for more **self-help offerings.** | | | |
| --- | --- | --- | --- |
| Yes 🞏 | How **important** is it to you? | | |
|  | **Very** important  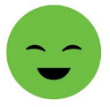🞏 | **Important**  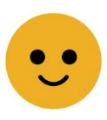🞏 | **Less** important  🞏  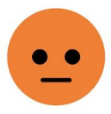 |
| No 🞏 | | | |

| 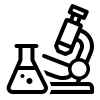  I wish for more **scientific** **research** about aphasia. | | | |
| --- | --- | --- | --- |
| Yes 🞏 | How **important** is it to you? | | |
|  | **Very** important  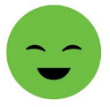🞏 | **Important**  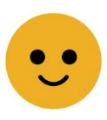🞏 | **Less** important  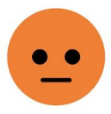🞏 |
| No 🞏 | | | |

| 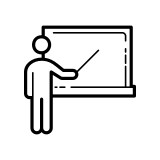  I wish for more **information** on the **results** of aphasia **research**. | | | |
| --- | --- | --- | --- |
| Yes 🞏 | How **important** is it to you? | | |
|  | **Very** important  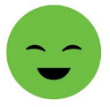🞏 | **Important**  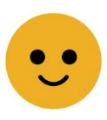🞏 | **Less** important  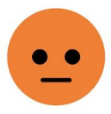🞏 |
| No 🞏 | | | |

| I wish for my **relatives** to be **involved** in speech therapy. 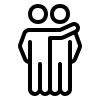 | | | |
| --- | --- | --- | --- |
| Yes 🞏 | How **important** is it to you? | | |
|  | **Very** important  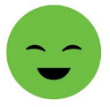🞏 | **Important**  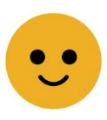🞏 | **Less** important  🞏  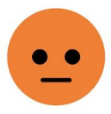 |
| No 🞏 | | | |

| 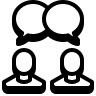  I wish to **have a say** in determining the **contents** of speech therapy. | | | |
| --- | --- | --- | --- |
| Yes 🞏 | How **important** is it to you? | | |
|  | **Very** important  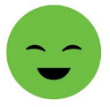🞏 | **Important**  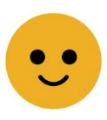🞏 | **Less** important  🞏  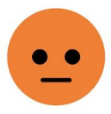 |
| No 🞏 | | | |

| 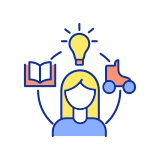  I wish for my **interests** to be **considered** in speech therapy. | | | |
| --- | --- | --- | --- |
| Yes 🞏 | How **important** is it to you? | | |
|  | **Very** important  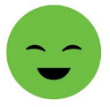🞏 | **Important**  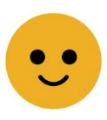🞏 | **Less** important  🞏  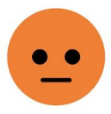 |
| No 🞏 | | | |

| 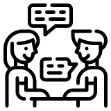  I wish for more **information** about the **effectiveness** of speech therapy **exercises**. | | | |
| --- | --- | --- | --- |
| Yes 🞏 | How **important** is it to you? | | |
|  | **Very** important  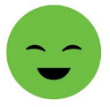🞏 | **Important**  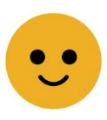🞏 | Less important  🞏  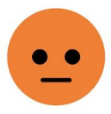 |
| No 🞏 | | | |

| 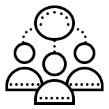I wish for there to be **communication** **between** my s**peech therapist in the practice** and the s**peech therapists in the rehabilitation clinics** regarding my **progress**. | | | |
| --- | --- | --- | --- |
| Yes 🞏 | How **important** is it to you? | | |
|  | **Very** important  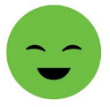🞏 | **Important**  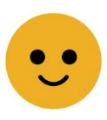🞏 | **Less** important  🞏  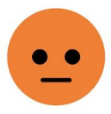 |
| No 🞏 | | | |

| 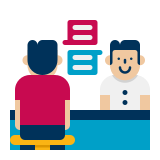  I wish for a counseling center for my condition. | | | |
| --- | --- | --- | --- |
| Yes 🞏 | How **important** is it to you? | | |
|  | **Very** important  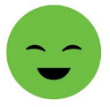🞏 | **Important**  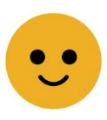🞏 | **Less** important  🞏  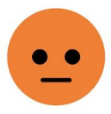 |
| No 🞏 | | | |

| 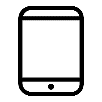  I wish for more **digital** **therapy** **options**. | | | |
| --- | --- | --- | --- |
| Yes 🞏 | How **important** is it to you? | | |
|  | **Very** important  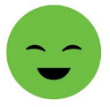🞏 | **Important**  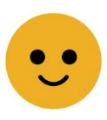🞏 | **Less** important  🞏  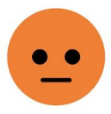 |
| No 🞏 | | | |

| 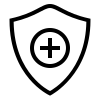  I wish for more **support** from the **health** **insurance** **company.** | | | |
| --- | --- | --- | --- |
| Yes 🞏 | How **important** is it to you? | | |
|  | **Very** important  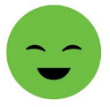🞏 | **Important**  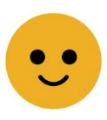🞏 | **Less** important  🞏  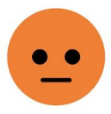 |
| No 🞏 | | | |

| 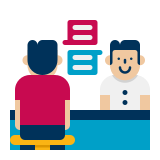  I wish for someone to **accompany** me **through** my **illness**. | | | |
| --- | --- | --- | --- |
| Yes 🞏 | How **important** is it to you? | | |
|  | **Very** important  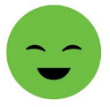🞏 | **Important**  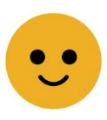🞏 | **Less** important  🞏  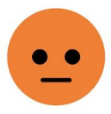 |
| No 🞏 | | | |

| Are there any other wishes regarding the care of people with aphasia? Do you want to tell us other reasons why you are not receiving any or sufficient speech therapy? |
| --- |
|  |
|  |
|  |
|  |

The following sections includes questions about yourself:

| How **old** are you? | | 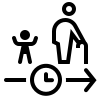 | | | | | | ________ Years | | |
| --- | --- | --- | --- | --- | --- | --- | --- | --- | --- | --- |
|  | | | | | | | | | | |
| Which **gender** do you identify with? | | | | | | | | | | |
| 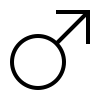male  🞏 | | | | 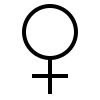female  🞏 | | | | 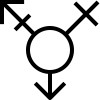diverse  🞏 | | |
|  | | | | | | | | | | |
| **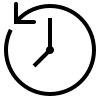Since** **when** do you have aphasia? | | | | | Answer: | | | | | |
|  | | | | | | | | | | |
| 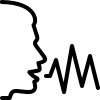  How **severe** do you **estimate** your aphasia to be? | | | | | | | | | | |
| mild  🞏  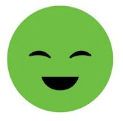 | | | | moderate  🞏  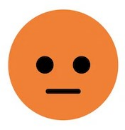 | | | severe  🞏  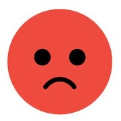 | | | |
|  | | | | | | | | | | |
| 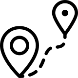  How far is the **nearest** speech therapy? | | | | | | | | | | |
| 0 – 30 minutes  🞏 | | | | 31 – 60 minutes  🞏 | | | More than 60 minutes  🞏 | | | |
|  | | | | | | | | | | |
| **Where** do you **live**? | | | | | | | | | | |
| 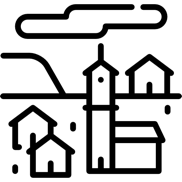Village  🞏 | | 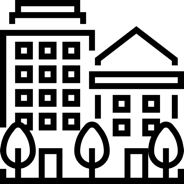 small town  🞏 | | | | | | **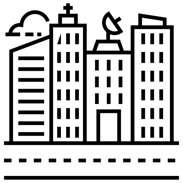**big city  🞏 | | |
| 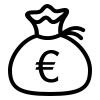How is your **financial** **situation**? | | | | | | | | | | |
| I am doing **very** **well**. | I am doing **well**. | | I am doing **just** **fine**. | | | I have to **restrict** myself. | | | | I have to **severly** **restrict** myself. |
| 🞏  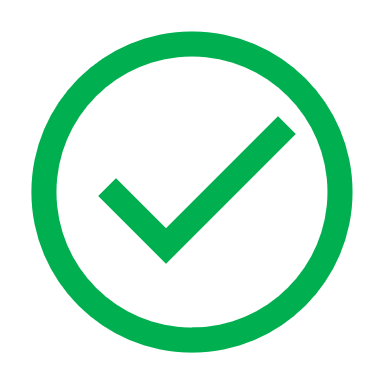 | 🞏  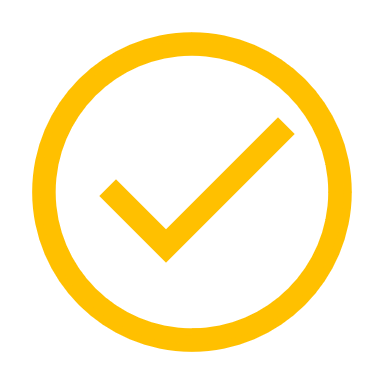 | | 🞏  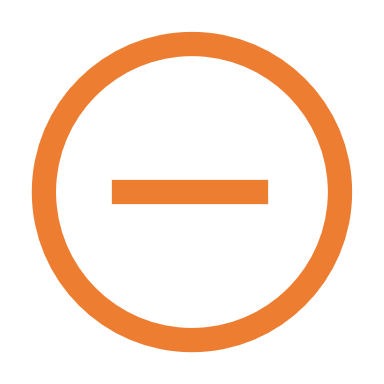 | | | 🞏  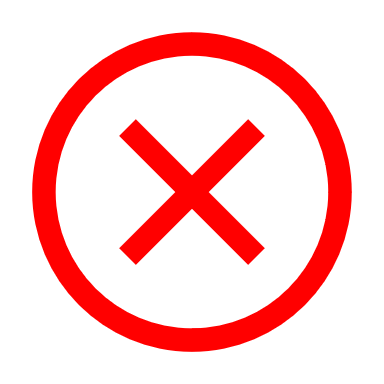 | | | | 🞏 |
|  | | | | | | | | | | |
| What professional qualification do you have? | | | | | | | | | | |
| **No** professional qualification  🞏 | | | | **Completed** professional qualification  🞏 | | | | | **University degree**  🞏 | |
|  | | | |  | | | | |  | |
| In which **country** do you live? | | | | | | | | | | |
| Germany  🞏 | | | | Austria  🞏 | | | | | Switzerland  🞏 | |

The next section present questions about your personality. Please chose the appropriate option.

| I see myself as someone who is **reserved**. | | |  | | |
| --- | --- | --- | --- | --- | --- |
| 🞏 | 🞏 | 🞏 | | 🞏 | 🞏 |
| **Strongly**  agree | **Agree** | **Neither** agree or disagree | | **Disagree** | **Strongly** disagree |

| I see myself as someone who is **generally** **trusting**. | | |  | | |
| --- | --- | --- | --- | --- | --- |
| 🞏 | 🞏 | 🞏 | | 🞏 | 🞏 |
| **Strongly**  agree | **Agree** | **Neither** agree or disagree | | **Disagree** | **Strongly** disagree |

| I see myself as someone who **tends** **to** **be** **lazy**. | | |  | | |
| --- | --- | --- | --- | --- | --- |
| 🞏 | 🞏 | 🞏 | | 🞏 | 🞏 |
| **Strongly**  agree | **Agree** | **Neither** agree or disagree | | **Disagree** | **Strongly** disagree |

| I see myself as someone who is **relaxed**, **handles** **stress** **well**. | | | |  | |
| --- | --- | --- | --- | --- | --- |
| 🞏 | 🞏 | 🞏 | 🞏 | | 🞏 |
| **Strongly**  agree | **Agree** | **Neither** agree or disagree | **Disagree** | | **Strongly** disagree |

| I see myself as someone who has **few artistic interests**. | | | | |
| --- | --- | --- | --- | --- |
| 🞏 | 🞏 | 🞏 | 🞏 | 🞏 |
| **Strongly**  agree | **Agree** | **Neither** agree or disagree | **Disagree** | **Strongly** disagree |

| I see myself as someone who is **outgoing**, **sociable**. | | | | |
| --- | --- | --- | --- | --- |
| 🞏 | 🞏 | 🞏 | 🞏 | 🞏 |
| **Strongly**  agree | **Agree** | **Neither** agree or disagree | **Disagree** | **Strongly** disagree |

| I see myself as someone who **tends** **to** **find faults with others**. | | | | |
| --- | --- | --- | --- | --- |
| 🞏 | 🞏 | 🞏 | 🞏 | 🞏 |
| **Strongly**  agree | **Agree** | **Neither** agree or disagree | **Disagree** | **Strongly** disagree |

| I see myself as someone who does a **thorough** **job**. | | | | |
| --- | --- | --- | --- | --- |
| 🞏 | 🞏 | 🞏 | 🞏 | 🞏 |
| **Strongly**  agree | **Agree** | **Neither** agree or disagree | **Disagree** | **Strongly** disagree |

| I see myself as someone who gets **nervous** **easily**. | | | | |
| --- | --- | --- | --- | --- |
| 🞏 | 🞏 | 🞏 | 🞏 | 🞏 |
| **Strongly**  agree | **Agree** | **Neither** agree or disagree | **Disagree** | **Strongly** disagree |

| I see myself as someone who has an **active** **imagination**. | | | | |
| --- | --- | --- | --- | --- |
| 🞏 | 🞏 | 🞏 | 🞏 | 🞏 |
| **Strongly**  agree | **Agree** | **Neither** agree or disagree | **Disagree** | **Strongly** disagree |

Is there anything else you would like say?

|  |
| --- |
|  |
|  |
|  |
|  |
|  |
|  |
|  |

Thank you for participating!
