# Supplementary material for: Influence of the Big Five personality traits on intensive therapy adherence within people with aphasia
Source: Front Public Health. 2026 Mar 30;14:1793090. doi: 10.3389/fpubh.2026.1793090 (PMC13071038; doi:10.3389/fpubh.2026.1793090)
Supplement: Supplementary file 3 [file Supplementary_file_3.docx]

|  | **Big Five Personality Traits** | | | | | | | | | | | | | | | | | | | | | | |
| --- | --- | --- | --- | --- | --- | --- | --- | --- | --- | --- | --- | --- | --- | --- | --- | --- | --- | --- | --- | --- | --- | --- | --- |
|  | **Extraversion** | | | **Agreeableness** | | | | | **Consciousness** | | | | | **Neuroticism** | | | | | **Openness** | | | | |
| **Reasons for non-guideline-based speech therapy** | *U* | *Z* | *p* | *U* | | *Z* | | *p* | *U* | | *Z* | | *p* | *U* | | *Z* | | *p* | *U* | | *Z* | | *p* |
| Speech therapist nearby only treat children. | 2283.5 | -.825 | .409 | 2068.5 | | -.1534 | | .125 | 2533.5 | | -.013 | | .990 | 2164 | | -1.214 | | .225 | 2451 | | -.282 | | .778 |
| My speech therapist said that I do not longer need therapy. | 2122.5 | -1.246 | .213 | 1839.5 | | -2.175 | | .**030** | 2145.5 | | -1.177 | | .239 | 1925 | | -1.886 | | .059 | 2385 | | -.399 | | .690 |
| My speech therapist does/did not suit me. | 1947 | -.276 | .783 | 1774.5 | | -.904 | | .366 | 1906.5 | | -.424 | | .672 | 1763.5 | | -.938 | | .348 | 2016 | | -.027 | | .978 |
| I do/did not like the goals set in speech therapy. | 2395.5 | -.064 | .949 | 2346.5 | | -.227 | | .820 | 2309.5 | | -.349 | | .727 | 2226 | | -.623 | | .533 | 2332 | | -.275 | | .784 |
| It is/was too exhausting. | 1854.5 | -.296 | .767 | 1930 | | -.019 | | .985 | 1866.5 | | -.253 | | .800 | 1327.5 | | -2.238 | | .**025** | 1600.5 | | -1.237 | | .216 |
| Speech therapy is too expensive. | 3392.5 | -1.402 | .161 | 3923.5 | | -.032 | | .974 | 3576 | | -.934 | | .351 | 3485 | | -1.165 | | .244 | 3444.5 | | -1.274 | | .203 |
| I have no time for speech therapy. | 1501 | -1.936 | .053 | 1578.5 | | -1.669 | | .095 | 2013.5 | | -.104 | | .917 | 1578 | | -1.661 | | .097 | 1924 | | -.425 | | .671 |
| There are no speech therapists nearby. | 2962.5 | -.724 | .469 | 3147 | | -.198 | | .843 | 3139 | | -.221 | | .825 | 3112 | | -.297 | | .766 | 3036 | | -.516 | | .606 |
| My aphasia is not severe enough. | 2044.5 | -1.795 | .*073* | 2028.5 | | -1.857 | | .063 | 2244.5 | | -1.169 | | .242 | 2248 | | -1.153 | | .249 | 2192 | | -1.335 | | .182 |
| I have had aphasia for a long time. | 4307.5 | -.097 | .923 | 3824.5 | | -1.286 | | .199 | 3550 | | -1.958 | | .050 | 4291.5 | | -.136 | | .892 | 4181.5 | | -.407 | | .684 |
| I have no one who supports me. | 1655 | -.770 | .441 | 1483 | | -1.420 | | .155 | 1438.5 | | -1.585 | | .113 | 1695 | | -.621 | | .534 | 1606.5 | | -.955 | | .340 |
| It is stressful to constantly get prescriptions from the doctor. | 3331.5 | -.004 | .997 | 3084.5 | | -.698 | | .485 | 3312.5 | | -.058 | | .954 | 3051.5 | | -.787 | | .432 | 3304.5 | | -.080 | | .936 |
| My doctor does not longer prescribe speech therapy. | 2520.5 | -.532 | .595 | 2617 | | -232 | | .816 | 2498 | | -.605 | | .545 | 2600 | | -.284 | | .776 | 2612 | | -.248 | | .804 |
| I do not make any progress anymore. | 2828.5 | -.508 | .611 | 2970.5 | | -.092 | | .927 | 2457.5 | | -1.605 | | .109 | 2669 | | -.977 | | .329 | 2903.5 | | -.289 | | .773 |
|  | | | | | | | | | | | | | | | | | | | | | | | |
| **Other factors** | *r* | *p (2-tailed)* | *p (1-tailed)* | *r* | *p (2-tailed)* | | *p (1-tailed)* | | *r* | *p (2-tailed)* | | *p (1-tailed)* | | *r* | *p (2-tailed)* | | *p (1-tailed)* | | *r* | *p (2-tailed)* | | *p (1-tailed)* | |
| Therapy frequency | .079 | .227 | .112 | .081 | .215 | | .107 | | .212** | <.**001** | | <.**001** | | -.115* | .077 | | .**038** | | -.138* | .**033** | | .**017** | |
| Therapy frequency satisfaction | .001 | .988 | .494 | -.001 | .989 | | .494 | | .021 | .748 | | .374 | | -.020 | .695 | | .349 | | .009 | .892 | | .446 | |
|  | | | | | | | | | | | | | | | | | | | | | | | |
